# The association between platelet glycoprotein-specific antibodies and response to short-term high-dose dexamethasone with prednisone maintenance treatment in adult patients with primary immune thrombocytopenia

**DOI:** 10.1080/07853890.2021.2018486

**Published:** 2022-01-04

**Authors:** Yan-Qiu Hou, Yan Wang, Chang-Xun Liu, Shu-Xia Li, Ya-Lan Peng, Wang Dong-Dong, Ru-La Sa

**Affiliations:** Department of Blood, Hulunbeir People's Hospital, Hulunbeir, China

**Keywords:** Primary immune thrombocytopenia, platelet glycoprotein-specific autoantibody, treatment response, dexamethasone, prednisone

## Abstract

**Objective:**

The aim of the present study was to detect the association between platelet glycoprotein-specific autoantibodies and the patient response to short-term high-dose dexamethasone (HD-DXM) + prednisone maintenance treatment.

**Methods:**

The data from 112 adult patients newly diagnosed with ITP who were administered first-line HD-DXM + prednisone maintenance therapy between January 2016 and January 2021 were retrospectively analyzed.

**Results:**

A total of 72 patients positive for platelet glycoprotein-specific antibodies were enrolled in the antibody-positive group, and 40 patients not positive for platelet glycoprotein-specific antibodies were enrolled in the antibody-negative group. In the antibody-positive group, six platelet glycoprotein-specific antibody types were found: 41.67% of the patients were anti-GP IIb/IIIa-positive only, 5.56% were anti-GP Ib/IX-positive only, 5.56% were anti-P-selectin-positive only, 19.44% were anti-GP IIb/IIIa- and anti-GP Ib/IX-positive, 16.67% were anti-GP Ib/IX- and P-selectin-positive and 11.11% were positive for all three antibodies. There was no significant difference in the overall response rate between the antibody-positive group and the antibody-negative group (94.44 versus 80.00%, *p* = .221). However, the CR rate was significantly higher in the antibody-positive group than in the antibody-negative group (69.44% versus 40.00%, *p* = .032). The logistic regression analysis revealed that platelet glycoprotein-specific antibody positivity and age were two factors that could affect patient response.

**Conclusions:**

The present study discovered that adult patients newly diagnosed with ITP who had positive platelet glycoprotein-specific antibody test results were likely to achieve a better response after treatment with HD-DXM + prednisone maintenance.

## Introduction

Primary immune thrombocytopenia (ITP) is a common haematologic disorder affecting patients of all ages [1,2]. It is characterized by isolated thrombocytopenia (platelet count <100 × 10^9^/L) and mucocutaneous bleeding resulting from an autoimmune condition in which platelets are destroyed by immune-mediated mechanisms [3]. Other than primary ITP, different diseases or disorders, such as systemic lupus erythematosus, hepatitis C infection and lymphoproliferative disorders, can also result in thrombocytopenia [4]. In clinical practice, the diagnosis of primary ITP is based principally on the exclusion of other ITP causes [4,5].

Steroids and intravenous immunoglobulin (IVIG) are recommended as the first-line treatment methods for primary ITP [6]. Short-term high-dose dexamethasone (HD-DXM) (40 mg/day × 4 days) or long-term conventional-dose prednisone (1 mg/kg/day) are commonly used steroid treatment protocols [7]. However, almost one-third of patients do not respond to steroid treatment. Of the adult patients with ITP who responded to steroid therapy, 30–50% could not achieve sustained response after steroid interruption [7,8]. Thus, steroid treatment methods for primary ITP are being challenged.

Previous studies have proposed that prolonging the steroid exposure time may help obtain sufficient immunosuppression for patients with ITP [7]. Din et al. conducted a study comparing the efficacy of HD-DXM alone or combined with low-dose DXM maintenance in patients with ITP [9] and found that HD-DXM + low-dose DXM was an effective treatment protocol for patients with unresponsive ITP [9]. Xu et al. conducted a real-world study demonstrating that HD-DXM + prednisone maintenance achieved a good efficacy in patients with newly diagnosed ITP [7].

Platelet glycoprotein-specific antibodies play an important role in the pathogenesis of ITP [10]. The autoantibodies can bind to the circulating platelets, resulting in the reticuloendothelial system clearing the platelet [10]. These platelet autoantibodies include anti-glycoprotein (GP) IIb/IIIa, GP Ib/IX and P-selectin. Autoantibodies against GP IIb/IIIa and GP Ib/IX have been detected in 70–80% and 20–40% of patients with ITP, respectively [11]. Platelet glycoprotein-specific autoantibodies have been used to distinguish patients with primary ITP from patients without ITP who have thrombocytopenia [12]. Furthermore, the expressions of platelet glycoprotein-specific autoantibodies could be used to predict the efficacy of treatment protocols. For example, patients with ITP who had anti-GP Ib/IX antibodies were found to be less responsive to IVIG treatment [13], and patients with ITP who had anti-GP Ibα antibodies or antibodies against both GP Ibα and GP IIb/IIIa showed a low steroid response [14]. However, it has not yet been determined if these platelet glycoprotein-specific autoantibodies could affect the response of patients with ITP to the treatment protocol of HD-DXM + prednisone maintenance.

Thus, this study aims to detect the association between platelet glycoprotein-specific autoantibodies and patient response to the treatment protocol of HD-DXM + prednisone maintenance.

## Materials and methods

### Patients

Between January 2016 and January 2021, our hospital conducted a series of observational studies to evaluate the efficacy of HD-DXM + prednisone maintenance in the treatment of primary ITP. In this observational cohort study, the data from adult patients newly diagnosed with ITP, who were hospitalized in the haematology units, were retrospectively analyzed. The study was approved by the institutional review board of our hospital. All enrolled patients signed an informed consent form.

Inclusion criteria: (1) patients who met the International Working Group diagnostic criteria for primary ITP [15]; (2) patients aged ≥18 years; (3) patients newly diagnosed with ITP; and (4) patients who underwent the platelet glycoprotein-specific antibody test before treatment. Exclusion criteria: patients with secondary, relapsed or refractory ITP.

The enrolled patients were divided into two groups according to the platelet glycoprotein-specific antibody test results: either antibody-positive or antibody-negative groups.

### Treatment methods

All patients newly diagnosed with ITP who were enrolled in this study received the treatment protocol of intravenous HD-DXM (40 mg/day × 4 consecutive days) immediately after the diagnosis. These patients then received oral prednisone (1 mg/kg/day) followed by 0.2 mg/kg/week over a 6-week period. In patients with severe ITP who had clinically important bleeding manifestations, high-dose IVIG (0.4 g/kg/day × 3–5 days) was used.

### Data collection

Patient data, including age, gender, height, weight, treatment protocol, ITP-specific bleeding score (bleeding symptoms were scored according to the ITP bleeding scale) [16], expression of platelet glycoprotein-specific antibodies, treatment response and adverse reactions were retrospectively collected.

The complete blood count, including the platelet count, was measured by a haematology analyzer (Mindray Biomedical Electronics Co., Ltd., Shenzhen, China). The serum platelet glycoprotein-specific antibodies were determined using enzyme-linked immunosorbent assay kits in accordance with the manufacturer’s instructions (Assaypro LLC, Saint Charles, MO).

The patient response within three months of the treatment onset was evaluated as complete response (CR), response (R) or no response (NR). CR was defined as a platelet count ≥100 × 10^9^/L without bleeding manifestations; R was defined as a platelet count ≥30 × 10^9^/L or a two-fold increase over baseline platelet count without bleeding; and NR was defined as a platelet count <30 × 10^9^/L or < two-fold increase of baseline platelet count or bleeding. The overall response = CR + R, and the time to response (TTR) was defined as time from treatment onset to response.

### Statistical analysis

The SPSS 22.0 software (SPSS Inc., Chicago, IL, USA) was used to analyze the data. Normal distribution quantitative data were described as mean ± standard deviation and compared using Student’s *t* test; non-normal distribution quantitative data were described as median with interquartile range and compared with the Mann–Whitney *U* test; and categorical data were described as numbers and percentages and compared using the Chi-square test. The difference in treatment response between patients with and without platelet glycoprotein-specific antibodies were compared using the Chi-square test, and the logistic regression analysis was performed to quantify the effect of variables on overall response. A *p* value of <.05 indicated statistical significance.

## Results

### Patient characteristics

A total of 112 newly diagnosed ITP patients with a median age of 61 (27–75) years were retrospectively studied. Among them, 52 (46.43%) were men and 60 (53.57%) were women. The median platelet count before treatment was 13 × 10^9^/L (0–30 × 10^9^/L). A total of 72 patients were enrolled in the antibody-positive group, while 40 patients were enrolled in the antibody-negative group. The characteristics of patients in both groups are shown in Table 1. No significant differences were observed in age, gender, platelet count before treatment, bleeding score, body mass index (BMI), IVIG use and comorbidities between the two groups (*p* > .05 in all).

**Table 1.** Patient characteristics.

Characteristics	Antibody positive group (*n* = 72)	Antibody negative group (*n* = 40)	*p* Value
Median age (y) (range)	64 (32–75)	60 (29–74)	.739
Male/female, *n*	34/38	18/22	.821
Median platelet count (×10^9^/L) (range)	12.5 (1–29)	18.5 (0–30)	.095
Median bleeding score (range)	1 (0-8)	1 (0-8)	.914
BMI (kg/m^2^)	23.22 ± 3.43	23.17 ± 3.10	.954
Combined with IVIG, *n* (%)	30 (41.67)	13 (32.50)	.339
Comorbidities at diagnosis, *n* (%)			
Diabetes	8 (11.11)	5 (12.50)	.930
Hypertension	27 (37.50)	11 (27.50)	.284
Thyroid disease	5 (6.94)	3 (7.50)	.785
Coronary heart disease	11 (15.28)	6 (15.00%)	.969
Antiplatelet antibodies, *n* (%)			
Anti-GP IIb/IIIa positive	52 (72.22)	–	–
Anti-GP Ib/IX positive	38 (52.78)	–	–
Anti-P selectin positive	24 (33.33)	–	–

In the antibody-positive group, 30 (41.67%) patients were anti-GP IIb/IIIa-positive only; 4 (5.56%) were anti-GP Ib/IX-positive only; 4 (5.56%) were anti-P-selectin-positive only; 14 (19.44%) were anti-GP IIb/IIIa- and anti-GP Ib/IX-positive; 12 (16.67%) were anti-GP Ib/IX- and P-selectin-positive; and 8 (11.11%) were positive for all three antibodies.

### Treatment response

Among all the patients with ITP, 68 (60.71%) achieved CR, 32 (28.57%) achieved R and 12 (10.71%) did not respond to treatment. The overall response rate was 89.29%. Of the 112 patients who received HD-DXM + prednisone maintenance therapy, 43 (38.39%) also received high-dose IVIG. As shown in Table 2, there was no significant difference in the overall response between the antibody-positive group and the antibody-negative group (94.44 versus 80.00%, *p* = .221). However, the CR rate was significantly higher in antibody-positive groups than in antibody-negative groups (69.44 versus 40.00%, *p* = .032). Through further analysis, it was found that patients who were anti-GP IIb/IIIa-positive had a higher CR rate when compared to patients who were antibody-negative (80.33 versus 40.00%, *p* < .001). There was no significant difference in the CR rate between patients with the other five types of antibodies and patients who were antibody-negative (*p* > .05). The median TTR was 5 (1–16) days in the antibody-positive group and 6.5 (2–26) days in the antibody-negative group. There was no statistical difference in TTR between the two groups (*p* = .357).

**Table 2.** Response to treatment in antibody positive and negative patients.

	CR, *n* (%)	R, *n* (%)	NR, *n* (%)	Overall response rate (%)
Antibody positive group (*n* = 72)	50 (69.44)*	18 (25.00)	4 (5.56)	94.44
Anti-GP IIb/IIIa (+) only	24 (80.00)*	6 (20.00)	0 (0)	100.00
Anti-GP Ib/IX (+) only	2 (50.00)	2 (50.00)	0 (0)	100.00
Anti-P selectin (+) only	2 (50.00)	2 (50.00)	0 (0)	100.00
Anti-GP IIb/IIIa (+) plus anti-GP Ib/IX (+)	10 (71.43)	0 (0)	4 (28.57)	71.43
Anti-GP Ib/IX (+) plus anti-P selectin (+)	8 (66.67)	4 (33.33)	0 (0)	100.00
Anti-GP IIb/IIIa (+) plus anti-GP Ib/IX (+) plus anti-P selectin (+)	4 (50.00)	4 (50.00)	0 (0)	100.00
Antibody negative group (*n* = 40)	16 (40.00)	16 (40.00)	8 (20.00)	80.00

CR: complete response; R: response; NR: no response; *indicated that *p* < .05 versus antibody negative group.

The univariate and multivariate logistic regression analyses revealed that gender, BMI, platelet count before treatment and IVIG use were not associated with the overall response of patients with ITP (Table 3). Platelet glycoprotein-specific antibody positivity and age were two factors that could affect patient’s overall response. The multivariate logistic regression analysis showed that the odds ratio (OR) of overall response for antibody-positive patients to antibody-negative patients was 4.320 (*p* = .034, 95% CI: 1.113–16.767, Table 3), indicating that antibody-positive patients were prone to better treatment response.

**Table 3.** The effect of baseline characteristics on treatment response by logistic regression analysis.

Variable	Univariate OR (95% CI)	*p* Value	Multivariate OR (95% CI)	*p* Value
Platelet glycoprotein-specific antibody	4.278 (1.314–13.928)	.016	4.320 (1.113–16.767)	.034
Gender	2.017 (0.656–6.197)	.221	2.690 (0.708–10.224)	.146
Age	1.052 (1.005–1.010)	.031	1.061 (1.007–1.118)	.027
BMI	0.924 (0.779–1.097)	.368	0.877 (0.713–1.079)	.216
Platelet count before treatment	0.952 (0.893–1.015)	.130	0.970 (0.898–1.047)	.436
The use of IVIG	2.283 (0.630–8.272)	.209	1.808 (0.391–8.372)	.449

OR: odd ratio; CI: confidence interval.

### Safety

No patients died within three months of the treatment onset. No femoral head necrosis, Cushing face or edoema were found during the follow-up period. A total of 13 (11.61%) patients developed hypertension after treatment. Of them, 8 patients were in the antibody-positive group and 5 were in antibody-negative group; the difference between the two groups was not significant (*p* = .930). A total of 8 patients developed hyperglycaemia after treatment. Of them, five patients were in the antibody-positive group and three were in the antibody-negative group; the difference between the two groups was not significant (*p* = .913).

## Discussion

In the present study, it was found that patients newly diagnosed with ITP who were platelet glycoprotein-specific antibody-positive after receiving treatment protocol of HD-DXM + prednisone maintenance had a higher CR rate than patients who were platelet glycoprotein-specific antibody-negative. The logistic regression analysis revealed that antibody-positive patients were prone to a better treatment response.

Platelet glycoprotein-specific antibodies play an important role in dictating ITP therapy response. A Chinese study conducted by Liu et al. found that patients with ITP who had anti-GP IIb/IIIa antibodies had a good response to corticosteroids (long-term conventional-dose prednisone) [17]. Chen et al. found that GP IIb/IIIa antibody-positive patients had a high response to DXM treatment, while GP Ibα antibody-positive patients had a worse response [18]. A study conducted by Zeng et al. also found that patients with anti-GP IIb/IIIa antibodies had a higher steroid response [14]; however, this response was lower in patients with anti-GP Ibα antibodies or antibodies against both GP Ibα and GP IIb/IIIa [14].

In contrast with the above-listed studies, the present study used the protocol of HD-DXM + prednisone maintenance to treat patients newly diagnosed with ITP. Compared with the commonly used steroid treatment protocols (HD-DXM or long-term conventional-dose prednisone), HD-DXM + prednisone maintenance prolonged the steroid exposure time, obtaining more sufficient ITP immunosuppression. In a study conducted by Xu et al., 70 out of 72 patients achieved R and 76.4% achieved a sustained response after treatment with HD-DXM + sequential prednisone, indicating that the use of this treatment protocol as the first-line treatment for patients newly diagnosed with ITP could achieve good clinical efficacy [7].

Anti-GP IIb/IIIa and anti-GP Ib/IX are two common primary autoantibodies found in patients with ITP [11]. The P-selectin autoantibody is another important platelet glycoprotein-specific antibody that may affect the functions of platelets and endothelial cells [19]. Previous studies have found that the platelet destruction methods of anti-GP IIb/IIIa antibodies and anti-GP Ib/IX antibodies are different: the former is mainly dependent on the Fc pathway, and the latter mainly clears the platelet via the Fc-independent way [20]. Thus, patients who are anti-GP IIb/IIIa-positive may have a different treatment response than patients who are anti-GP Ib/IX-positive.

Previous studies have indicated that patients with anti-GP IIb/IIIa antibodies had a good response to steroids, while patients who were positive for anti-GP Ibα or two antibodies did not respond to steroid treatment well [17]. In this study, the proportions of patients with anti-GP Ib/IX and anti-P-selection antibodies were low. Thus, the treatment response could not be compared among patients with different antibody types. However, it was found that antibody-positive patients had a significantly higher CR rate than antibody-negative patients. Furthermore, among the six antibody types, only patients with anti-GP IIb/IIIa achieved a significantly higher CR rate than antibody-negative patients. These results indicated that antibody-positive patients achieving a high CR rate mainly depended on the presence of anti-GP IIb/IIIa; this is consistent with the results of other studies [14,18]. The logistic regression analysis showed that platelet glycoprotein-specific antibody positivity and age were two factors that could affect the patient response to the protocol of HD-DXM + prednisone maintenance. The OR of the response in antibody-positive patients to antibody-negative patients was as high as 4.320. This result further indicated that antibody-positive patients were prone to a better treatment response.

Xu’s study found that the morbidity and severity of steroid-related adverse events of HD-DXM + prednisone maintenance treatment were within an acceptable range [7]. The present study also revealed that this treatment protocol was safe. No patients died, and no severe adverse events were found during the follow-up period. Furthermore, the incidence of adverse events, such as hypertension and hyperglycaemia, was not significantly different in antibody-positive and antibody-negative patients.

The present study has several limitations. First, the sample size was small, and all patients were from a single centre. This may cause selection bias. Second, the proportions of patients with anti-GP Ib/IX and anti-P-selection antibodies were low. Hence, the treatment response could not be compared among patients with different antibody types. Furthermore, the observation period was short, and the long-term efficacy of the treatment protocol of HD-DXM + prednisone maintenance in patients with ITP who were either platelet glycoprotein-specific antibody-positive or negative was not investigated. Finally, the reason patients with ITP who were platelet glycoprotein-specific antibody-positive could achieve a higher response rate was not determined. Thus, further confirmatory studies with a larger number of patients are necessary.

## Conclusion

In conclusion, the present study discovered that adult patients newly diagnosed with ITP who had positive platelet glycoprotein-specific antibody test results were likely to achieve a better response after treatment with HD-DXM + prednisone maintenance.

## Data Availability

All data generated or analyzed during this study are included in this published article.
